# Texture and Microstructural Features at Different Length Scales in Inconel 718 Produced by Selective Laser Melting

**DOI:** 10.3390/ma12081293

**Published:** 2019-04-19

**Authors:** Michele Calandri, Shuo Yin, Barry Aldwell, Flaviana Calignano, Rocco Lupoi, Daniele Ugues

**Affiliations:** 1 Dipartimento di Scienza Applicata e Tecnologia (DISAT), Politecnico di Torino, Corso Duca degli Abruzzi, 24, 10129 Turin, Italy; michele.calandri@polito.it; 2 Department of Mechanical and Manufacturing Engineering, Trinity College Dublin, The University of Dublin, Parsons Building, Dublin 2, Ireland; yins@tcd.ie (S.Y.); aldwellb@tcd.ie (B.A.); lupoir@tcd.ie (R.L.); 3 Nammo Ireland, 15–17 Northwest Business Park, Ballycoolin, Dublin 15, Ireland; 4 Dipartimento di Ingegneria Gestionale e della Produzione (DIGEP), Politecnico di Torino, Corso Duca degli Abruzzi, 24, 10129 Turin, Italy; flaviana.calignano@polito.it

**Keywords:** selective laser melting, Inconel 718, crystallographic texture, subgranular dendrites, epitaxial growth

## Abstract

Nickel-based Inconel 718 is a very good candidate for selective laser melting (SLM). During the SLM process, Inconel 718 develops a complex and heterogeneous microstructure. A deep understanding of the microstructural features of the as-built SLM material is essential for the design of a proper post-process heat treatment. In this study, the microstructure of as-built SLM Inconel 718 was investigated at different length scales using optical microscopy (OM), scanning electron microscopy (SEM), and transmission electron microscopy (TEM). Electron backscatter diffraction (EBSD) was also used to analyze the grain morphology and crystallographic texture. Grains elongated in the build direction and crossing several deposited layers were observed. The grains are not constrained by the laser tracks or by the melt pools, which indicates epitaxial growth controls the solidification. Each grain is composed of fine columnar dendrites that develop along one of their <100> axes oriented in the direction of the local thermal gradient. Consequently, prominent <100> crystallographic texture was observed and the dendrites tend to grow to the build direction or with occasional change of 90° at the edge of the melt pools. At the dendrite length scale, the microsegregation of the alloying elements, interdendritic precipitates, and dislocations was also detected.

## 1. Introduction

Among all the nickel-based superalloys, Inconel 718 is one of the most studied alloys, which has been used in a wide range of industrial sectors, such as aeronautical, aerospace, and energy production. Inconel 718 possesses excellent mechanical properties, creep- and fatigue-resistance performance at high temperature [1,2], and hot-oxidation resistance [3,4]. Components manufactured from Inconel 718 often operate in highly aggressive environments at temperatures higher than 700 °C, or at cryogenic temperatures, such as in jet engines, gas turbine engines, chemical and nuclear plants, heat exchangers, and cryogenic tanks [5,6,7,8]. Inconel 718 is a ternary Ni-Cr-Fe system and is composed of a solid solution austenitic γ strengthened by very small and finely-dispersed precipitates of intermetallic phases γ′-Ni_3_(Ti,Al) and γ″-Ni_3_Nb [9]. This alloy is conventionally produced through casting followed by cold working [5,10], and is one of the most weldable nickel superalloys due to its low strain-age cracking sensitivity during the post-weld heat treatment [11,12,13]. In particular, this low susceptibility to cracking makes Inconel 718 a good candidate [14] for additive manufacturing (AM).

During the last decade, the fabrication of Inconel 718 alloy through AM has gained more and more attention [15,16,17,18,19,20] from both the scientific and industrial communities. AM techniques allow the customized production of near net-shape dense metal parts with complex geometries. Therefore, the use of expensive tools, dies, or casting molds can be avoided [21,22]. Among all AM processes, selective laser melting (SLM) is an interesting technique that uses high power-density laser as a heat source to induce local melt of a metallic powder bed. The melt pool then consolidates through rapid solidification, and the fabrication of SLM components is achieved through the addition and melting of several layers of metal powders [23,24]. SLM can produce close to fully dense Inconel 718 components with mechanical properties, after appropriate heat treatment, comparable to and even better than cast or wrought counterparts [25,26].

During the SLM process, the material being built will be rapidly melted by the laser and will then solidify under very high cooling rates and directional heat fluxes, which have a significant effect on the microstructure evolution and mechanical properties [27,28,29,30,31]. In addition, the solidified material is subject to repeated thermal cycles during the deposition of the following layers [32], which further complicates the microstructure evolution because of the partial dissolution of the second phases formed during the solidification, the redistribution of the solutes, the precipitation of new phases [33], and the increased heterogeneity of the microstructure that results from the aforementioned phenomena [34]. Therefore, SLM Inconel 718 alloy in the as-built state will often possess a very complex microstructure characterized by anisotropy [35], strong crystallographic texture [34,35,36], microsegregation, and the formation of second phases [17,37,38]. These microstructural features can significantly affect the material mechanical properties before and after heat treatments [25,38,39,40]. Therefore, a comprehensive understanding of the evolution of the microstructure during SLM is essential for the application of SLM technology to the fabrication of Inconel 718 and other nickel-based superalloys.

Although Inconel 718 alloy produced through SLM process has been extensively studied [41,42], a complete and detailed study of the microstructure and the pre-existing second phases in the as-built state focusing on all different length scales is still lacking. In the present study, the microstructure of Inconel 718 produced through a SLM process was investigated at different length scales, starting from 10^−3^ to 10^−5^ m, within which laser-related features (i.e., laser track and melt pool boundaries) and grains with characteristic crystallographic texture could be observed, to 10^−5^–10^−6^ m, within which intragranular substructures consisting of fine cellular dendrites could be observed, and finally to 10^−6^–10^−8^ m, within which single dendrite and intercellular precipitates could be observed. Furthermore, the relationship between microstructure and solidification conditions is also discussed in this work. The aim of this study is to clearly identify, at all length scales, the main microstructural features derived directly from the SLM process without any post-process heat treatments. This basic knowledge is fundamental to determine if the as-built condition can eventually be suitable in principle to guarantee mechanical performance and high temperature hot oxidation resistance, and how the as-built microstructure can be corrected to optimize these properties with dedicated subsequent thermal treatments.

A preliminary study of the microstructural evolution of the SLM Inconel 718 alloy during the post-process heat treatment cycle is also reported in this paper. The effect of the solution annealing step on the as-built microstructure has been studied in past work by the authors, with an optimal homogenization/solution recipe of 1065 °C for 2 h identified [43]. Starting from this solutioned condition, analysis of the most important thermodynamic transformations that can occur during the aging process is provided.

## 2. Materials and Methods 

### 2.1. Manufacturing Procedure

**Figure 1:**
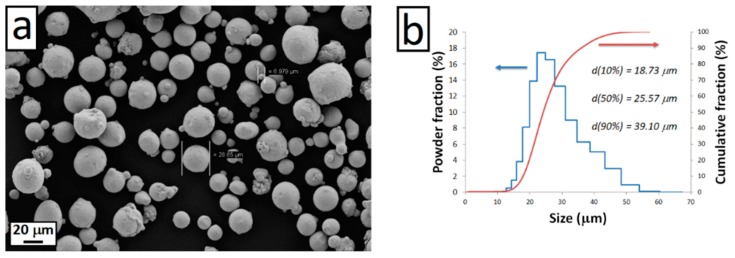
Surface morphology (**a**) and size distribution (**b**) of the Inconel 718 powders used in this work.

The SLM Inconel 718 samples studied in this work were manufactured using an EOSINT M270 dual mode machine (EOS GmbH, Krailling, Germany) equipped with 200W Yb fiber continuous laser beam. The feedstock was gas atomized Inconel 718 powders (EOS GmbH). The chemical composition of the Inconel 718 powder used in this work, according to the powder supplier’s specification, is listed in Table 1. Figure 1 shows the surface morphology and size distribution of the Inconel 718 feedstock powders. The powder was spherical in shape with small particles agglomerated on the surface of larger ones. A bidirectional scanning strategy was adopted for producing the Inconel 718 samples. The scanning trajectory was rotated by an angle of 67° between adjacent layers in order to reduce the anisotropy of the samples.

The samples were manufactured on the build platform directly without any supports. After manufacture, the samples were removed from the build platform by using electrical discharge machining.

### 2.2. Processing Parameters Optimization Procedure 

Before microstructural characterization, an optimization study of the main process parameters (i.e., laser power, scan speed, and hatching distance) was performed in order to identify the optimum parameters to produce a fully dense part. For this scope, a full factorial design of the experiment involving three levels for each parameter was used. A list of the experiments carried out and the parameter levels used in each is shown in Table 2. For each experiment a 15 mm × 15 mm × 10 mm cubic sample was produced. The spot size and the layer thickness were set to fixed values of 100 and 20 µm, respectively. In Table 2, the volumetric energy density (VED) value associated to each set of process parameters is also reported. VED (J/mm^3^) is as shown in Equation (1):

(1) $$VED=\frac{P}{vh_{d}d}$$

where P is laser power (W), v the scan speed (mm/s), $h_{d}$ the hatching distance (mm), and *d* is the layer thickness (mm).

The density level of each sample was evaluated through apparent density measurements, porosity evaluation through image analysis of optical micrographs (Leica DMI 5000 M optical microscope, Wetzlar, Germany), and Brinell hardness measurements. The apparent density was measured through the Archimedes method [45] using a precision balance (model bc, Orma s.r.l., Milan, Italy, resolution: 0.1 mg), with all samples being prepared for testing by polishing of all surfaces with SiC paper, in order to avoid measurement errors related to air trapping on the highly rough surface after the SLM production. The optical micrographs for the porosity evaluation were collected on samples previously polished with 1 µm diamond suspension, without any chemical etching. A total of 28 optical images were collected for each sample, each of them had an area of 1.8 mm × 1.4 mm (total area ≈ 73.6 mm^2^). The porosity level was calculated as a percentage of the analyzed surface area through the binarization of the micrographs by setting a threshold gray value; for each image, two threshold gray values were chosen in order to obtain a low estimate and a high estimate of porosity.

The average Brinell hardness of each sample was calculated from 5 indentations using an EMCO TEST M4U durometer (EMCO-TEST Prüfmaschinen GmbH, Kuchl, Austria), each indentation was performed by imposing a load of 62.5 kgf (≈ 613 N) for 15 s.

### 2.3. Microstructural Characterization of the As-Built State

For the microstructural characterization, cylindrical test samples of 15 mm in diameter and 125 mm in length were produced by adopting the optimal set of parameters previously identified and reported in Table 3. No stress relieving heat treatment was applied after the fabrication of these samples.

Metallographic samples were cut from the cylindrical bars along the direction perpendicular and parallel to the build direction (BD) to characterize the sample microstructure in the horizontal and vertical planes, respectively. The samples were prepared first with SiC grinding paper, and then polished with 1 µm diamond solution, and finally with 0.05 µm alumina suspension. Two etching techniques were used to reveal the microstructure: electrochemical etching using a solution of 100 mL HNO_3_ and 10 mL water with an imposed voltage of 1–2 V for about 3–4 s, and normal immersion etching by using waterless Kalling’s reagent (5 g CuCl_2_ in 100 mL HCl and 100 mL ethanol). The etched samples were then observed with an optical microscope (OM) equipped with a digital camera (Leica, Wetzlar, Germany) and with a scanning electron microscope (SEM, Carl Zeiss ULTRA, Oberkochen, Germany). 

The crystallographic texture was studied using electron backscattered diffraction (EBSD, Zeiss SUPRA 40, Oberkochen, Germany) equipped with a Bruker detector (Bruker Nano GmbH, Berlin, Germany). The samples for EBSD characterization were polished using standard polishing procedure followed by a long final polishing step with 0.05 µm alumina suspension. The EBSD analysis was carried out with a voltage of 20 kV and an exposure time of 150 to 200 ms. A total area of 215 µm × 170 µm and 150 µm × 190 µm was analyzed on the horizontal and vertical planes with a step size of 1.41 µm, respectively. A local area of 30 µm × 25 µm and 60 µm × 90 µm was also analyzed, respectively, on the horizontal and vertical planes with high-resolution (step size: 0.35 µm for the former and 0.7 µm for latter). The microstructure at the length scale of 10^−6^–10^−8^ m was characterized with scanning/transmission electron microscope (S/TEM, FEI Titan, Hillsboro, OR, USA) equipped with energy dispersive X-ray spectrometry (EDS) detector. The thin lamella for TEM observation was prepared at the vertical plane using focused ion beam (FIB, Carl Zeiss Auriga, Oberkochen, Germany) system.

### 2.4. Microstructural Evolution Investigation during Aging

Differential scanning calorimetry (DSC) was performed to determine the temperature ranges where the most important microstructural modifications occur. Cylindrical samples of 3.5 mm in diameter and 10 mm in height were produced through SLM, using the processing parameters reported in Table 3. A thermal analyzer, Setaram DSC/TGA 92 16.18 (Caluire, France), was used for the DSC analysis, with a heating rate of 20 °C/min, from room temperature to 1200 °C, in order to detect all the solid state transformations that occur both at the as-built condition and after a solution annealing at 1065 °C for 2 h.

On the basis of the DSC analysis, some temperatures of interest were identified. The microstructural modifications of the material were then investigated through progressive exposure to these temperatures. Small round plates (diameters: 13 mm, height: 3 mm) produced through SLM were used for this study: the samples were first treated by solution annealing at 1065 °C for 2 h and then aged following the recipes reported in Table 4. The samples were treated by inserting them into a preheated furnace room. At the end of the thermal exposure they were removed and cooled in still air; the small dimensions of the plate minimize the effects of the heating and cooling transients.

The Vickers microhardness of the aged samples was measured using a Vickers hardness indenter (Mitutoyo, Kawasaki, Japan) through a total of 10 microindentations for each sample. For comparison, the Vickers microhardness at the as-built state (14 microidentations) and after the 1065 °C/2 h solution annealing (10 microindentations) was also measured.

X-Ray diffraction (XRD) analysis was performed on the aged samples using a X-Pert Philips diffractometer (Amsterdam, The Netherlands) in Bragg–Brentano configuration emitting Cu Kα ration and scanning between 30° and 100° with a step size of 0.013°. Field emission scanning electron microscopy (FESEM) (Merlin Zeiss, Oberkochen, Germany and Carl Zeiss ULTRA) analysis was also used to evaluate the microstructural changes and the formed second phases.

## 3. Results

### 3.1. Optimization of the Process Parameters

**Figure 2:**
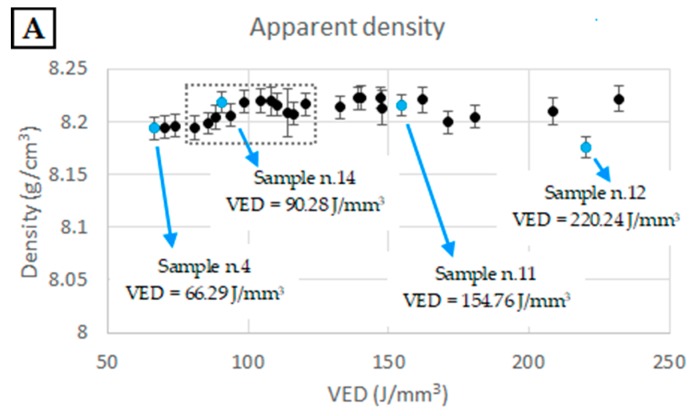

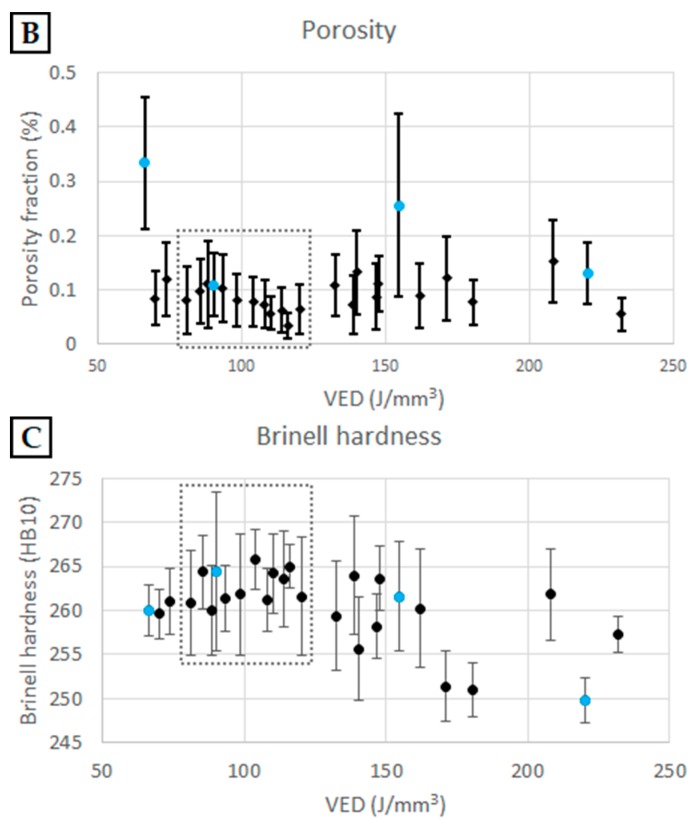
Apparent density (**A**), measured with the Archimedes method; porosity fraction (**B**), evaluated through optical images analysis; and Brinell hardness (**C**) of the selective laser melting (SLM) Inconel 718 samples, produced with different combinations of process parameters as functions of the volumetric energy density (VED) value. In each plot, the blue data points indicate samples n.4, n.14, n.11, and n.12; polished cross sections of which are shown in Figure 3 as examples. The error bars in plots A and C indicate 95% confidence intervals, while the bars in plot B indicate the ranges between the lower and higher estimates of the pore coverage ratio. Adapted from [46].

The samples obtained from the experiments listed in Table 2 were characterized through the measurement of the apparent density, porosity fraction, and Brinell hardness. The results of this preliminary study, reported in Figure 2 as functions of the VED value, show that the process was very robust, with no large variations in the porosity or hardness being observed within the investigated ranges. In particular, for all the combinations of process parameters, the density value fell within the range of 8.17 and 8.22 g/cm^3^ (theoretical maximum value) [8]. Furthermore, the porosities evaluated through image analysis of optical micrographs were always lower than 0.5%. Closer examination of the trends reported in Figure 2 shows that intermediate VED values tended to provide slightly better densification and hardness levels and lower variation of the data (i.e., higher process stability). The dotted boxes in the plots of Figure 2 indicate the optimum process window identified by this study.

**Figure 3:**
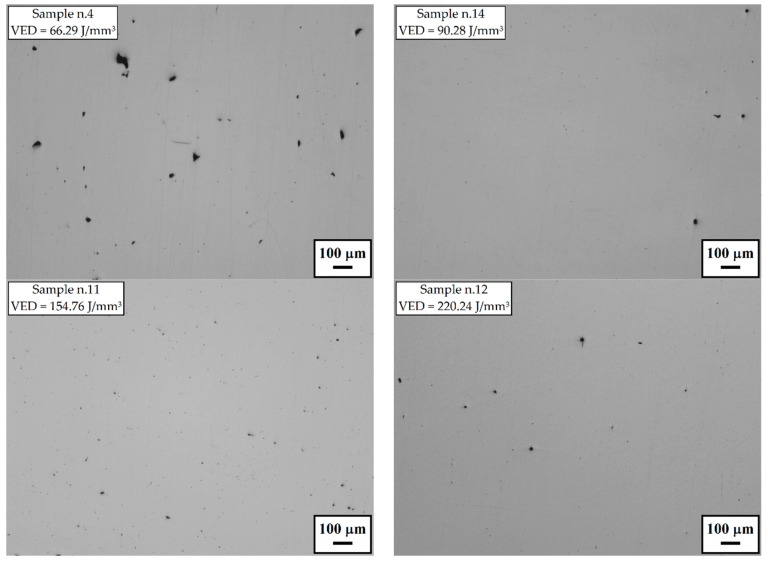
Some example optical micrographs showing the porosity of selective laser melting (SLM) Inconel 718 samples obtained with different volumetric energy density (VED) values. Sample n.14 was obtained with a VED value within the optimum processability window (dotted boxes in the plots of Figure 2).

Selective examples of the optical micrographs used for the porosity evaluation are shown in Figure 3. Sample n.14 is representative of the result that can be obtained with intermediate values of VED. Although some small pores of approximately 20 µm were still present, the evaluated porosity level was lower than 0.12% and the Brinell hardness fell into the range between 260 and 266 HB10.

From the micrographs shown in Figure 3, it is also possible to observe that for the sample manufactured with the lowest VED value the largest pores were observed (40–60 µm) with irregular shape due to lack of fusion. Conversely, higher VED values tend to form a slightly larger amount of spherical gas porosities compared to the ones in the optimum processability window. Furthermore, very high VED tends to reduce the Brinell hardness of the produced material.

The set of parameters used for sample n.14 (Table 3) were chosen for the microstructural characterization presented in the following subsections.

### 3.2. Grains and Laser-Related Microstructure (10^−^^3^–10^−^^4^ m)

**Figure 4:**
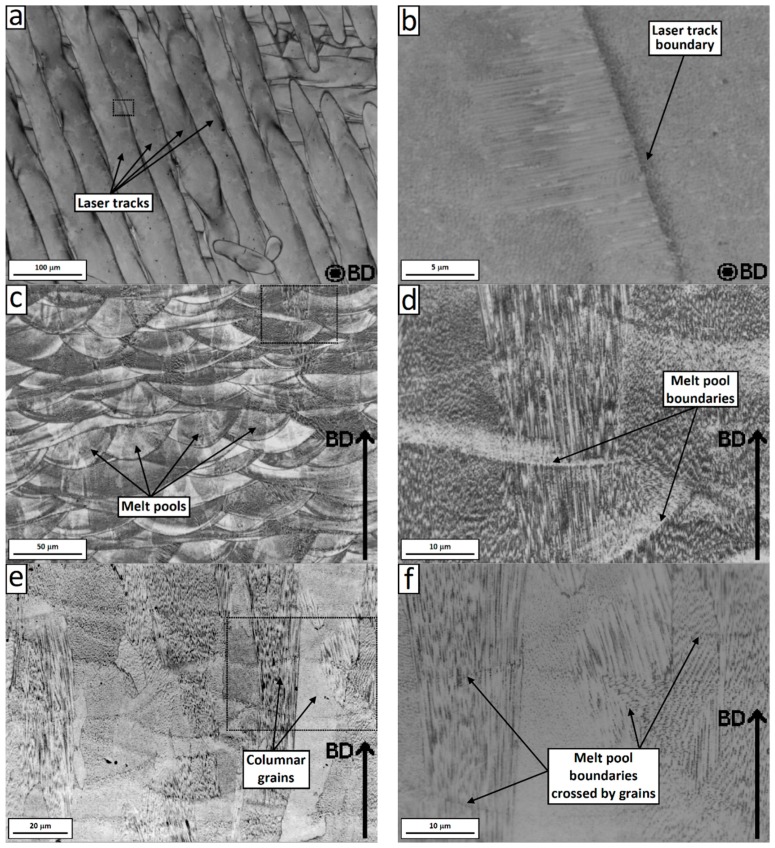
Optical Microscope (OM) micrographs of the electrochemically-etched SLM Inconel 718 on the horizontal plane with track–track MPBs and high magnification view (**a**,**b**), and vertical plane with arc-shape MPBs and high magnification view (**c**,**d**). OM micrographs of the Kalling solution etched Inconel 718 on the vertical plane showing columnar grains across several layers and high magnification view (**e**,**f**).

Figure 4a–d shows the OM micrographs of the electrochemically etched SLM Inconel 718 at the length scale of 10^−3^–10^−4^ m. As can be seen, the microstructure of the SLM Inconel 718 was characterized by two kinds of boundaries, which are laser related boundaries and grain boundaries. The traces of the laser passes are usually referred to as the track–track molten pool boundaries (MPBs) on the horizontal plane and the layer–layer MPBs on the vertical plane [17,47]. The former is related to the laser scanning strategy and hatch distance, while the latter has an arc shape and are created by the local melt boundaries during each laser pass. The grain boundaries were more clearly revealed after etching with Kalling’s reagent, as can be observed from the OM micrograph on the vertical plane shown in Figure 4e,f. It is interesting to observe that the grain boundaries were independent of the laser related boundaries. The grains tended to form an elongated shape oriented along the build direction. The grains spanned across several powder layers in the build direction. This shows that re-melting of subsurface layers, during subsequent laser passes, allows for the grain growth process to restart and span multiple layers. This creates strong bonds between the layers, reducing the risk of delamination and formation of inter-layer cracks [20].

**Figure 5:**
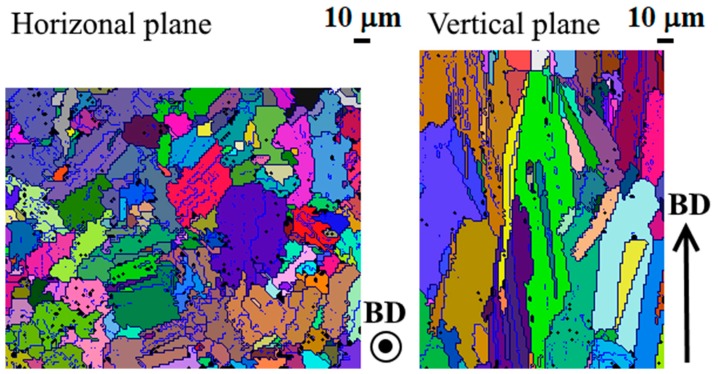
Grain maps of the horizontal and vertical planes of the selective laser melting (SLM) Inconel 718. The boundaries with a maximum misorientation angle of 10° or above are shown in dark blue, while the boundaries with a maximum misorientation angle between 4° and 10° are shown in lighter blue. The colors are used to distinguish the grains, with different shades of the same color indicating sub-granular crystalline domains within a single grain.

For further characterization of the grain structure of the SLM Inconel 718, Figure 5 shows EBSD maps of the horizontal and vertical planes. Note that only the matrix γ phase was detected with EBSD analysis. In the as-built state, the grains were characterized by the presence of subgranular domains, which are delimited by low-angle boundaries. In the grain maps shown in Figure 5, the high-angle boundaries between grains, which are defined as the boundaries with a maximum misorientation angle of 10° or higher, are pictured in dark blue; the boundaries with a maximum misorientation angle between 4° and 10° are depicted in lighter blue. Furthermore, the subgranular domains of each grain, surrounded by low-angle boundaries, are shown with different color shades. The largest grains contained five to 11 or even more subgranular domains. Note that the presence of preferred crystallographic orientations, which will be discussed in detail in Section 3.3, leads to some difficulties in identifying a clear misorientation threshold value to define the grain boundaries. The threshold values of 10° and 4° were chosen during the data processing step because they were found to best describe the grain structure.

It is clear that most of the grains sectioned on the horizontal plane appeared equiaxed, which means that there is no preferential growth direction in this plane (i.e., orthogonal to the build direction).

The average grain size (equivalent diameter) on the analyzed area of the horizontal plane was 10.9 µm, but the grains were very heterogeneous in size, with a standard deviation of 8.7 µm. There were also some large grains of up to 50 µm or greater. On the vertical plane, the grains appeared elongated with the major axis aligned with the build direction. The average grain height, calculated using only the grains that are completely contained in the analysis area, was 28.5 µm, but grains with length of about 180 µm could also be observed. There were a number of grains which have grown across more than one layer (20 µm) and even up to ten layers for the largest grains. The average grain aspect ratio, weighted on the grain sizes, was 5.4.

In addition, the EBSD mapping on the vertical plane also demonstrates that no crystallographic changes occurred between layers (i.e., at each deposition the crystallographic orientation of the underlying material was maintained).

### 3.3. Crystallographic Texture (10^−^^4^–10^−^^5^ m)

**Figure 6:**
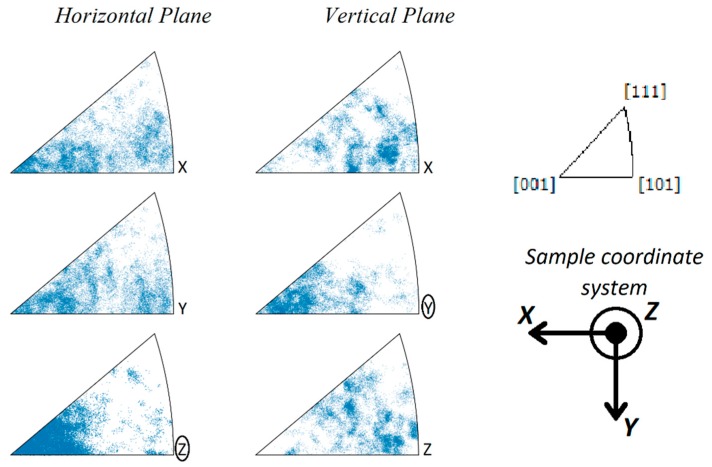
Inverse pole figure (IPF) charts obtained from electron backscatter diffraction (EBSD) analysis on the horizontal and vertical planes of SLM Inconel 718. [001] texture was detected on the IPF Z axis on the horizontal plane and on the IPF Y axis on the vertical plane.

The inverse pole figure (IPF) charts obtained from the EBSD analysis on the horizontal and vertical planes of the SLM Inconel 718 are shown in Figure 6, where a clear crystallographic texture was recognizable. On the horizontal plane, the detected points accumulated at the [001] vertex of the Z axis, with few detected points close to the [111] vertex of the X and Y axes. Similarly, on the vertical plane, the detected points accumulated at the [001] vertex of the Y axis, with few detected points close to the [111] vertex of the X and Z axes. Note that the build direction to the Z axis on the horizontal plane, and the Y axis on the vertical plane. The IPF charts can be interpreted as the preferential orientation of crystals, which tend to align their [001] direction along the build direction. Once the [001] axis is fixed, there still exists one degree of freedom for the orientation of the crystals, which is the Euler angle (ϕ_2_) of rotation around this axis. No preferential orientation related to ϕ_2_ can be deduced from the IPFs; therefore, no clear crystallographic anisotropy existed on the plane of the added layers.

**Figure 7:**
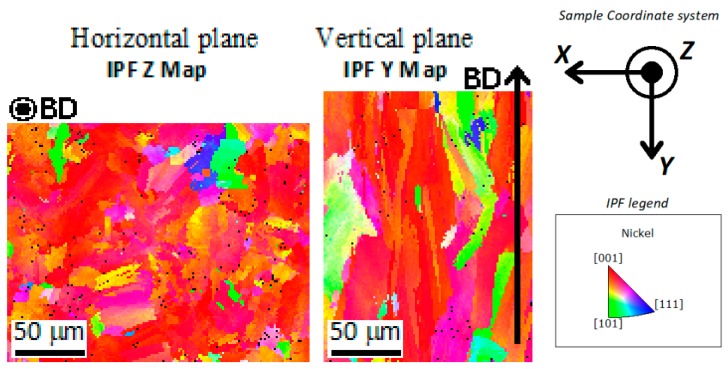
Inverse Pole Figure (IPF) maps of the Z axis for the horizontal plane and Y axis for the vertical plane showing grains with [001] orientation.

**Figure 8:**
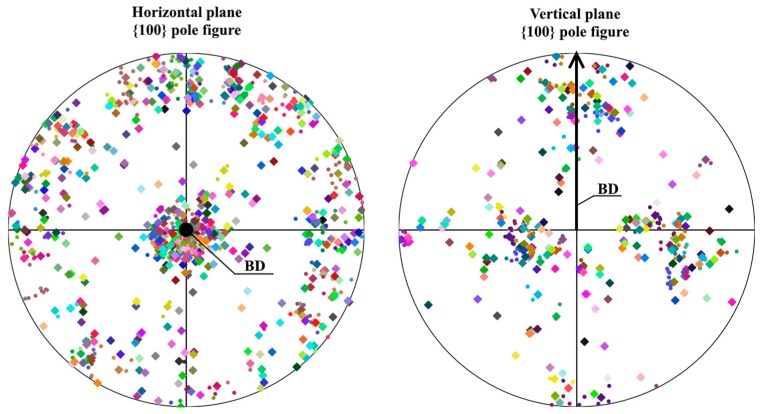
Pole figures of the horizontal and vertical planes of selective laser melting (SLM) Inconel 718. Each square represents the average orientation of the {100} crystallographic planes of the grains, as shown in the electron backscatter diffraction (EBSD) mapping in Figure 5, with the same colors used for each grain. The small circles represent the average orientation of the subgranular domains.

Figure 7 shows the IPF maps of the horizontal plane (vertical to the Z axis of the SCS) and vertical plane (vertical to the Y axis of the SCS). It is clear that most of the grains had their [001] crystallographic direction orientated close to or along the build direction. Figure 8 shows the pole figures at the {100} planes of the horizontal and vertical planes. Each large square indicates the average orientation of one of the grains shown in the maps in Figure 5. The small circles indicate the average orientation of the subgranular domains. It is clear that the [001] axis of most grains was close to the build direction, with no evident orientation around this axis.

**Figure 9:**
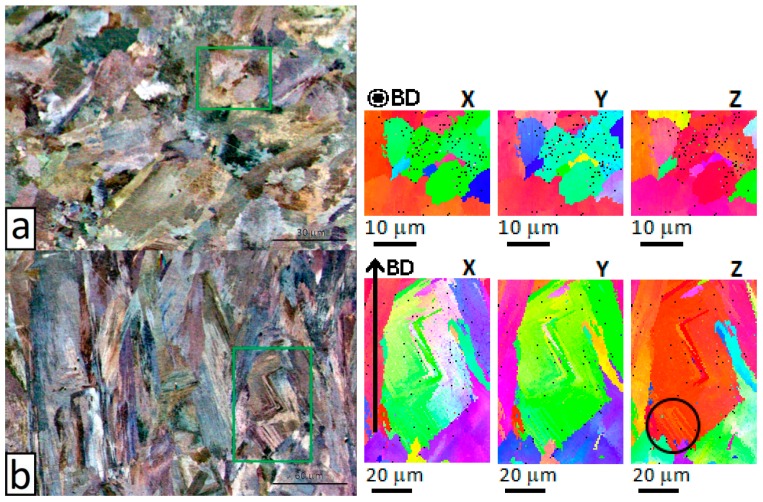
Forescattered/backscattered electron images on the horizontal plane (**a**) and vertical plane (**b**) with relative inverse pole figure (IPF) maps at higher resolution of the areas marked by the green boxes.

High-resolution EBSD analysis was also conducted on areas of interest in the horizontal and vertical planes. Figure 9 shows the relative IPF maps. The grains on the vertical plane had an interesting texture characterized by two subgrains, with zig-zag shape and a low angle (approximately 1°) boundary between each other. Dendrite substructure marked with a black circle can be observed in a grain on the vertical plane (Figure 9b), which will be further examined in the following section. No substructure was evident on the horizontal plane, with uniform crystallographic orientation inside each grain (Figure 9a).

### 3.4. Intragranular Dendrites (10^−^^5^–10^−6^ m)

**Figure 10:**
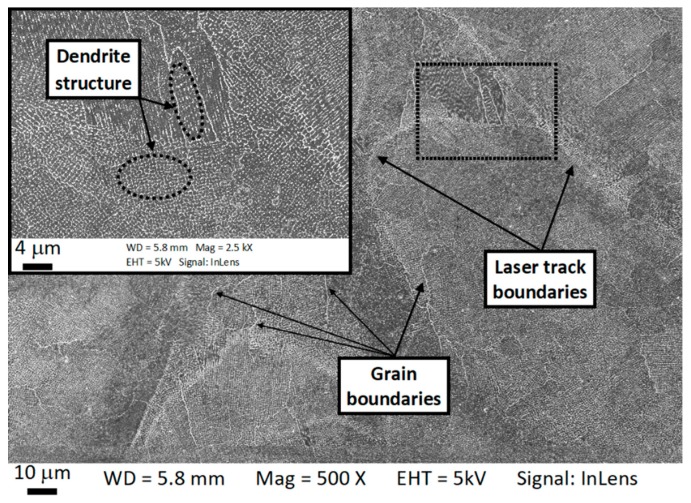
Scanning electron microscopy (SEM) micrographs of the horizontal plane showing laser tracks, grains and the subgranular dendrite structures.

**Figure 11:**
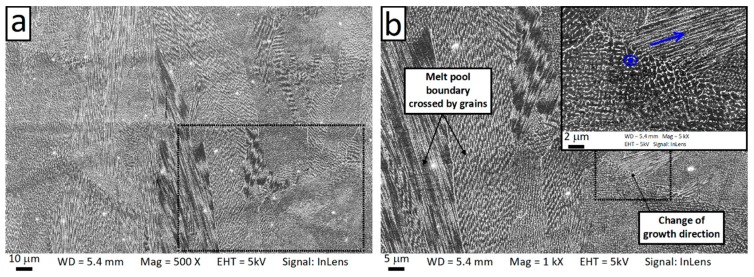
Scanning electron microscopy (SEM) micrographs of the vertical plane showing the columnar dendrites crossing melt pool boundaries (**a**) and the change of dendrite direction at the melt pool boundary (**b**). Blue arrows in (**b**) indicate the growth direction of the dendrites.

**Figure 12:**
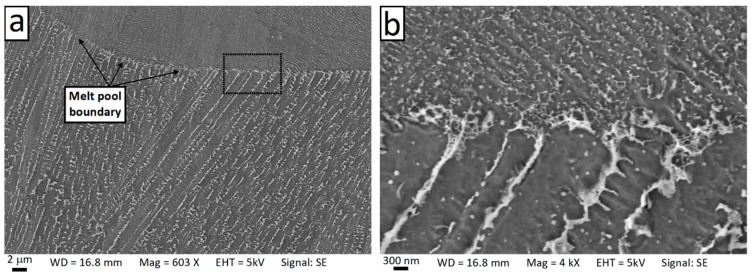
Scanning electron microscopy (SEM) micrograph of the vertical plane showing the change of the dendrite arm spacing and the growth direction when crossing a melt pool boundary. Panel (**b**) contains a higher magnification of the zone indicated by the dashed box in panel (**a**).

The internal microstructure of the grain was further investigated through SEM observation. At the length scale of 10^−5^–10^−6^ m, it is possible to observe the microstructure inside a single grain. Figure 10 and Figure 11 show SEM micrographs of the horizontal and vertical planes, respectively. Dendrites with short arm spacing and without secondary branching can be observed inside the grain. These dendrites had the same crystallographic orientation with very little to no misorientation between each other. In addition, it is also found that the dendrite arm spacing and direction were not homogeneous within the grain. This phenomenon can be clearly observed from Figure 11 and Figure 12, which show high-resolution SEM images of the boundary of a melt pool. As can be seen, abrupt changes of arm spacing and dentrite direction occured at the laser related boundaries. In particular, the dendrite size tended to be larger at the top of the melt pools and smaller at the bottom (Figure 12). Furthermore, the growth direction of the dendrites did not change when crossing a melt pool boundary in some cases, but rotateed by 90 degrees in other cases (Figure 11b). At the center of the laser tracks the dendrites developed along the build direction, while at the laser track boundaries they tended to rotate (this is also visible in Figure 4b).

### 3.5. Microsegregation, Interdendrite, and Intradendrite Phases (10^−6^–10^−8^ m)

**Figure 13:**
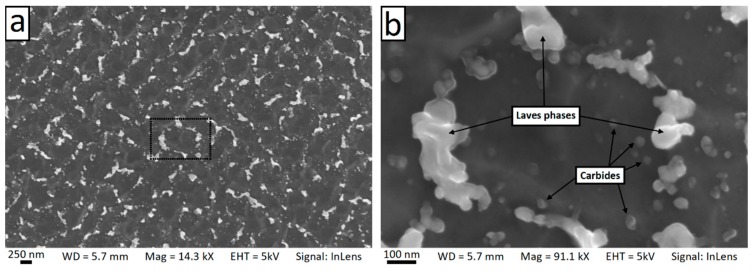
Scanning electron microscopy (SEM) micrographs showing the cellular microstructure on the horizontal plane (**a**) and high magnification view of a single dendrite (**b**).

**Figure 14:**
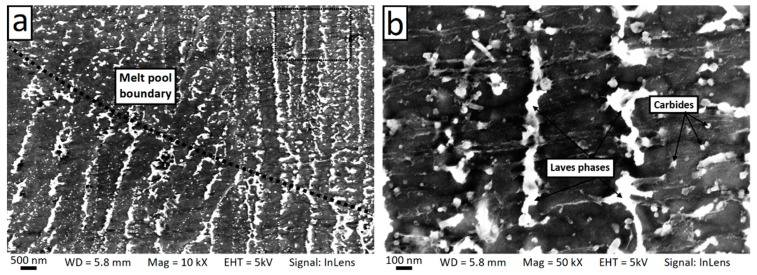
Scanning electron microscopy (SEM) micrographs showing the columnar dendrites on the vertical plane and the change in arm spacing across the melt pool boundary (**a**) and high magnification view (**b**).

Figure 13 shows SEM micrographs of the cross-section of dendrites (also known as the cellular structure) on the horizontal plane. It is clear that the dendrites exhibited a hexagonal pattern with an interspacing of 0.5–1 µm. In addition, a number of precipitates of second phases with a bimodal size distribution were visible. Irregularly-shaped precipitates of approximately 100–120 nm form at the boundaries of adjacent columnar dendrites. Furthermore, a large number of finer precipitates (25–50 nm) were also present in the intradendritic area. The density of the fine precipitates was higher near the edge of the dendrites. Figure 14 shows SEM micrographs of the columnar dendrites across a melt pool boundary on the vertical plane. Precipitates were clearly found around the dendrite boundaries.

**Figure 15:**
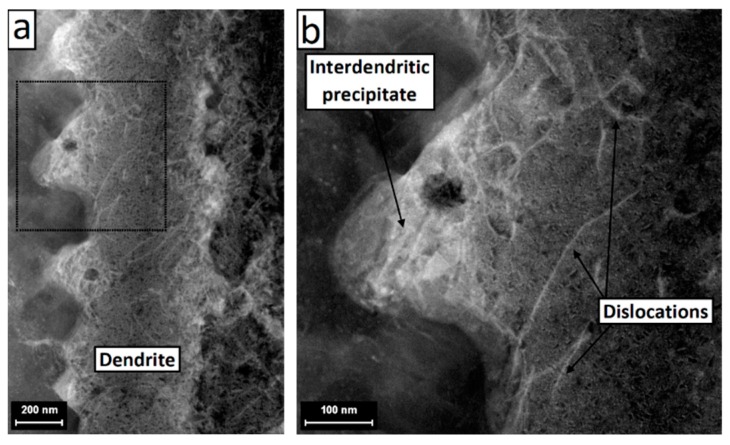
Scanning/transmission electron microscope (STEM) images of a dendrite (**a**) showing the dislocations and the interdendritic phases (**b**).

**Figure 16:**
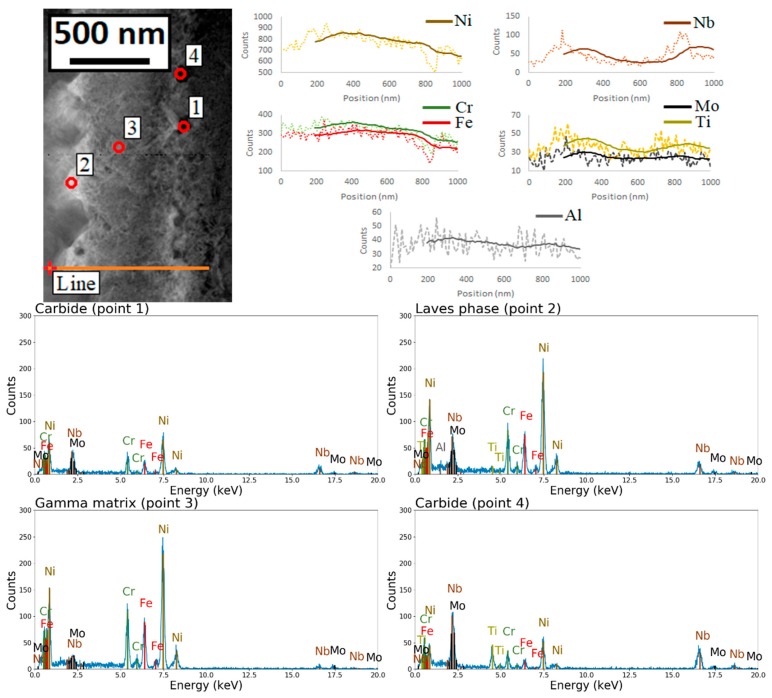
Energy dispersive X-ray spectrometry (EDS) line analysis across the dendrite showing microsegregation of the alloying elements and EDS analysis of interdendritic particles in comparison with the γ intradendritic phase (point 3). Point 2 was compatible with intermetallic Laves phase, while points 1 and 4 were likely carbides.

**Figure 17:**
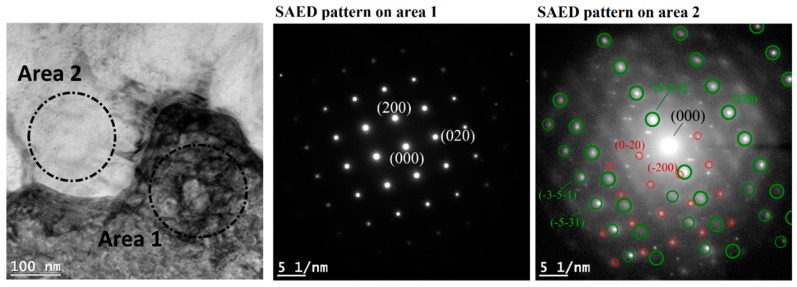
Transmission electron microscope (TEM) image showing the interface between γ phase (dark contrast) and an interdendritic precipitate (bright contrast) with respective selected area electron diffraction (SAED) patterns, both taken along the [001] zone axis of the γ phase. On the SAED pattern on area 2, the green circles underline diffraction points of Laves phase along its $\left[1\bar{1}2\right]$ zone axis, instead red circles underline diffraction points of the γ phase.

Figure 15 shows a scanning/transmission electron microscope (STEM) image of a single dendrite. High density dislocations were observed at the edge of the dendrite and around large interdendritic particles. Figure 16 shows an EDS line scan across the dendrite. Microsegregations of Nb, Mo, and Ti were detected at the edges of the dendrite. Ni, Cr, and Fe significantly decreased at one side of the dendrite where Nb content reacheed a peak. This suggests that different second phases were formed at each side of the dendrite. The EDS point analysis also indicates that, as compared with the matrix (point 3), point 1 was richer in Nb, point 2 was richer in Nb, Mo, and Ti, and point 4 was richer in Nb and Ti. Figure 17 shows the TEM image at the interface between the γ dendrite and an interdendritic particle. The selected area electron diffraction (SAED) patterns were taken along the [001] zone axis of the γ phase. It can be observed that the pattern detected on area 2 (i.e., on the interdendritic zone) was made by the superposition of the γ pattern, marked with red circles, and by a second pattern, marked with green circles, which can be interpreted as the $\left[1\bar{1}2\right]$ zone axis of the Laves phase, which has a hexagonal closely-packed structure and lattice parameters a = b = 0. 49 nm and c = 0. 78 nm [48,49]. The result of the SAED analysis suggests that a Laves/γ eutectic mixture was present in the interdendritic zone.

### 3.6. Aged State Microstructure

**Figure 18:**
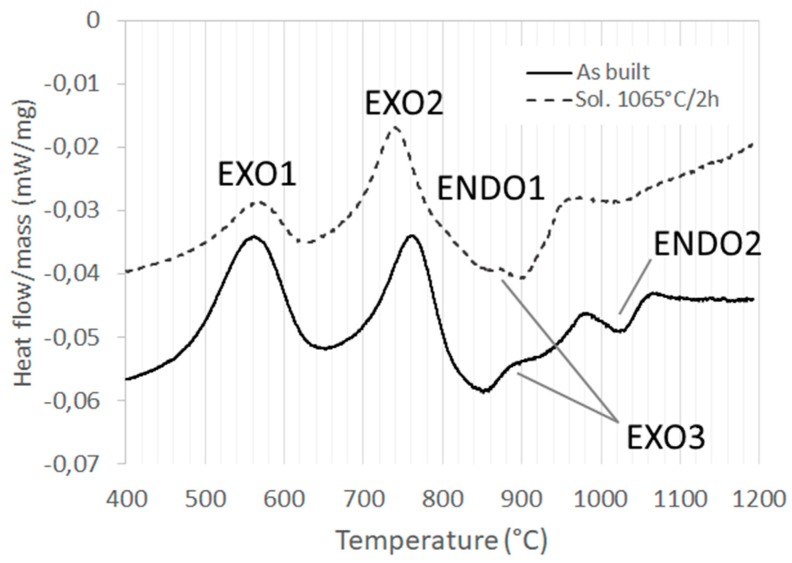
Differential scanning calorimetry (DSC) curves recorded on selective laser melting (SLM) Inconel 718 in the as-built state (full line) and solution heat treated at 1065 °C for 2 h (dotted line). The labels indicate the peaks related to exothermal (EXO) and endothermal (ENDO) phenomena.

The DSC curves obtained at the as-built and solutioned (1065 °C/2 h) conditions are reported in Figure 18, with the relative marked thermal phenomena occurred during the ramps. In both heating ramps, two exothermal signals (EXO1 and EXO2 peaks) were detected at 500–620 °C and 670–790 °C. Then, a wide endothermal signal (ENDO1) could be observed between 790 and 950 °C. In the ENDO1 temperature range, a third exothermal peak (EXO3) was detected at 850–910 °C. Finally, an endothermal signal (ENDO2) was present between 980–1070 °C. After the solution annealing, the detected signal was similar with respect to the as-built state; however, it was noted that the ENDO2 peak is much weaker.

**Figure 19:**
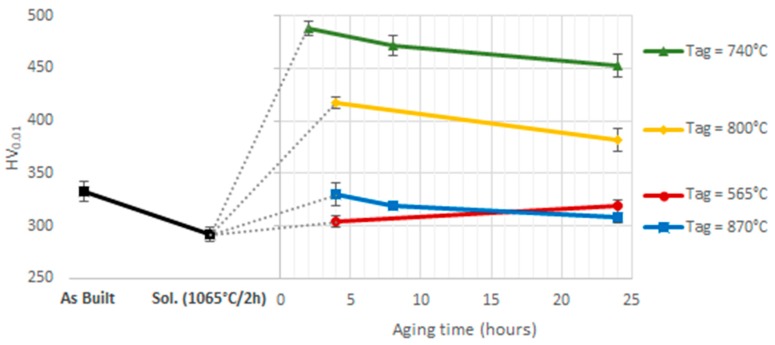
Vickers microhardness at different heat treatment conditions (as-built, solutioning, and aging at different temperatures and times).

Based on the DSC results from the solution heat-treated sample, the following temperatures were considered for the microstructural evolution during the aging step: 565 °C (EXO1 peak), 740 °C (EXO2 peak), 800 °C (EXO2 offset), and 870 °C (EXO3 peak). The mean Vickers microhardness measured on the aged samples are shown in Figure 19 in comparison with the as-built state and the 1065 °C/2 h solution heat-treated state. The solution heat treatment caused a 12.4% reduction of the Vickers microhardness due to the dissolution of most of the pre-existing second phases and the relieving of the residual stresses [50]. The aging treatment increased the Vickers microhardness of the alloy. Aging at 565 °C caused a slight hardening with respect to the solutioned condition; however, the mean Vickers microhardness was lower when compared to the as-built state, even after 24 h of aging. The greatest increase in hardness was obtained when aging at 740 °C, where a 46.6% increase of the Vickers microhardness, with respect to the as-built state, could be reached. The hardness reduced when the sample is aged for 24 h due to over aging. Aging at 800 °C was still able to improve the hardness of the as-built state, although the obtainable Vickers microhardness was lower as compared to that achieved after aging at 740 °C, especially when the treatment was prolonged to 24 h. After aging at 870 °C for 4 h, the Vickers microhardness was comparable to the as-built condition, with a slight decrease with longer treatment durations.

**Figure 20:**
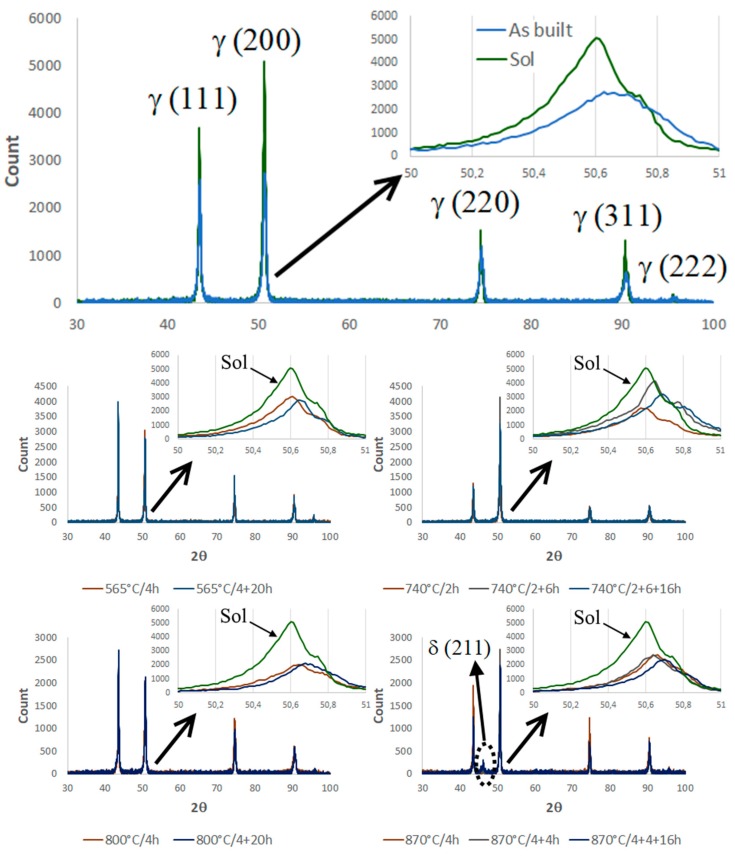
X-Ray diffraction (XRD) spectra acquired from SLM Inconel 718 samples at different heat treatment conditions (as-built, solutioning, and aging at different temperatures and times). The γ(200) peak is shown in higher resolution.

The XRD spectra are shown in Figure 20. In the majority of cases, only the γ matrix peaks were detected because there were insufficient amounts of the second phases to be detected by the XRD apparatus (carbides and Laves phases) or their peaks are overlapped with the γ peaks, as in the case of γ′ and γ″ [19]. The only exception was the δ (211) peak observed in the samples aged at 870 °C, which was also reported by Cao et al. in their study [51].

However, the effect of the thermal treatments (solution and aging) can be indirectly measured though the shift of the γ peaks. For example, in Figure 20 the shift of the (200) peak is shown in the higher magnification panels. The shift of the 2θ position was due to a slight variation of the γ matrix lattice parameter, which was related to the amount of the solute dissolved in it. A similar variation of the lattice parameter after heat treatment has previously been reported by Zhang et al. [52].

The lattice parameters can be calculated from the peak 2θ positions through the Bragg equation:

(2) nλ=2dsinθ

where λ is the wavelength used, d is the interplanar distance and θ is the reflection angle.

**Figure 21:**
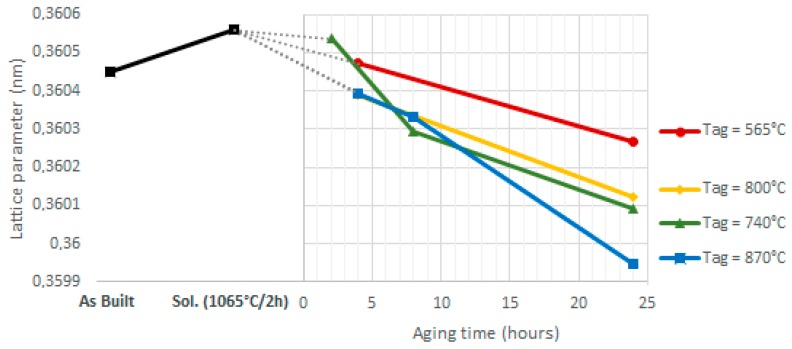
Lattice parameter of the γ matrix calculated from the X-Ray diffraction (XRD) spectra of Figure 20 with the Bragg equation. Trends at different heat treatment conditions (as-built, solutioning and aging at different temperatures and times).

The plot in Figure 21 shows the value of the γ lattice parameters after solution annealing at 1065 °C for 2 h followed by aging treatment, compared to the as-built condition. For each sample, the lattice parameter is the average of the interplanar distance values obtained from the γ(111), γ(200), γ(220), and γ(311) peaks using Equation (2). The cell parameter was obtained by averaging the values obtained from the first four peaks (the fifth is usually too weak).

**Figure 22:**
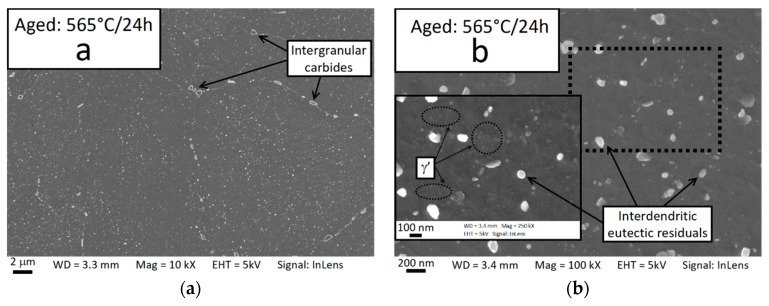

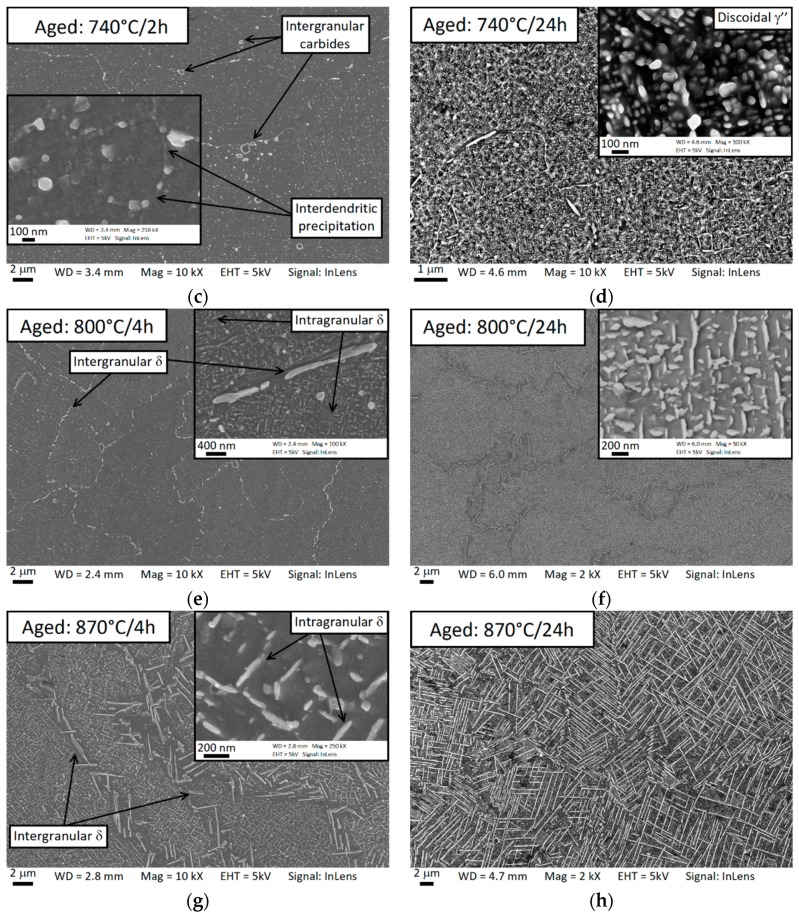
Field emission scanning electron microscopy (FESEM) micrographs showing the microstructure and the formation of precipitates after aging treatments of varying temperature and duration. Aging at 565 °C for 24 h (**a**) and higher magnification (**b**), aging at 740 °C for 2 h (**c**) and 24 h (**d**), aging at 800 °C for 4 h (**e**) and 24 h (**f**), aging for 870 °C for 4 (**g**) and 24 h (**h**).

Examples of FESEM micrographs collected for the solutioned and aged samples are shown in Figure 22. All micrographs show the horizontal plane. After the thermal treatment cycle, the as-built microstructure was significantly modified: The laser-related boundaries vanished, relatively large elongated or blocky precipitates (likely carbides [53]) formed at the grain boundaries, and the interdendritic Laves phases were dissolved so that only the smaller eutectic carbides remained as residuals. These observations are in agreement with Brenne et al. [50].

After aging at 565 °C for 4 h, no new second phases could be detected from the FESEM observation (not shown here for the sake of brevity); however, after 24 h very small particles (γ′ phase, see discussion) appeared in the intradendritic zone (see panel b of the 565 °C/24 h condition micrograph in Figure 22). Small intradendritic γ′ particles could be detected after aging at 740 °C for 2 h. Furthermore, a film-like precipitation occurred at the interdendritic boundaries where the Nb content was higher due to microsegregation. For longer durations, a high density of discoidal precipitates of about 35–65 nm (γ″ phase, see discussion) was observed.

During aging at 800 °C, the grain boundaries displayed plate-like δ precipitates and coarser discoidal γ″ precipitates formed in the intragranular zone. After 24 h aging at 800 °C, the intergranular plate-like precipitates were coarser and smaller plate-like δ precipitates were also formed within the grain.

The intergranular δ plates grew rapidly at 870 °C, furthermore a lot of plate-like intragranular precipitates were formed. The intragranular precipitates were 200–250 nm in length after 4 h of aging and 300–450 nm in length after 8 h (not included in the micrographs of Figure 22 for the sake of brevity). A large amount of very large plates (4–7 µm) uniformly dispersed across the metallographic surface was clearly visible after 24 h of aging at 870 °C.

## 4. Discussion

### 4.1. Considerations on the Grain Structure and Texture 

During the SLM process, materials are built through sequential solidification steps. At each pass, laser radiation causes an extremely fast melting of a local area of the powder layer and also part of the material that has already solidified during the previous passes [35], causing the formation of liquid volume. When the laser beam leaves the melt pool, the heat is rapidly released to the liquid–Ar atmosphere interface by convection and radiation and to the underlying substrate by conduction [54]. During this rapid cooling of the molten pool, solidification occurs predominantly through two competing phenomena: heterogeneous nucleation of new grains and epitaxial growth [55]. The epitaxial growth of the partially re-melted grains during the laser pass is a phenomenon widely reported in the literature [16,39,56,57] and causes the newly solidified material to inherit the crystallographic orientation of grains in contact with the liquid. Conversely, when heterogeneous nucleation occurs, a new grain with a random crystallographic orientation forms at the solid–liquid interface, interrupting the growth of the underlying grains.

The grain evolution during solidification depends on the crystallographic orientation with respect to the local thermal flux direction in the molten pool. The cubic crystals grow preferentially along the <100> directions [55,58]; therefore, those grains with a <100> axis oriented at a low angle with the local heat flux direction are favored (i.e., they grow faster and prevail over the others). Therefore, if some favorably oriented grains are present at the solid–liquid interface, these grains develop quickly, holding their crystallographic orientation through epitaxial growth and the frequency of formation of new randomly oriented grains through heterogeneous nucleation is low. Conversely, if the existing grains are not favorably oriented, their epitaxial growth is inhibited and thus the solidification of the molten volume occurs mainly through heterogeneous nucleation. When the epitaxial growth prevails, it leads to the formation of a small number of large grains, which are not confined by melt pools (i.e., independent from the laser related features) and a strong crystallographic texture is observed. When heterogeneous nucleation is the dominant solidification mechanism, many small grains confined in the melt pools and in the laser tracks are expected to form. The balance between epitaxial growth and heterogeneous nucleation depends on the thermal flux field in the molten pool, which in turn is influenced by the SLM process parameters and the adopted scan strategy. The exact form of the thermal flux field is usually very complex because of the influence of convective phenomena and the Marangoni effect [59,60]; however, at the solid–liquid interface the thermal fluxes tend to be aligned in a direction normal to the solidification front [61,62,63]. Therefore, at the boundaries between the laser tracks, the thermal gradients are oriented toward the center of the molten pool rather than along the build direction due to the arc shape of the melt pool [21,47,57].

When a bidirectional scan mode without rotation of the scan direction between successive layers is adopted, an alternated bands granularity is usually reported; this band structure is made of narrow columnar grains with a <100> axis predominately aligned to the build direction and elongated grains along the direction perpendicular to the scan direction, and with <100> and <110> texture along the scan direction and the build direction, respectively [63,64]. The band structure is related to the scan strategy, in particular to the repetition of the scanning pattern that makes the shape of the thermal flux field similar during deposition of each new layer. At the center of the laser scan path, the thermal fluxes have a strong component along the build direction that drives the epitaxial growth of the columnar grains, with [001] orientation created during the deposition of the previous layers. Instead, at the boundaries between the scan lines, the thermal fluxes are at an angle with respect to the build direction; therefore, the grains develop transversally in these zones through epitaxial growth, while heterogeneous nucleation prevails along the build direction. The elongated grains at the boundaries between the laser tracks tend to have a <100> axis aligned to the scan direction and the other two orthogonal <100> axes tilted at 45° respect to the build direction; in fact, this is the configuration that aligns the thermal flux field at the borders of the scan lines, and so grains nucleated with similar crystallographic orientations can grow faster and prevail over the others. If the scan direction is rotated by 90° between each new layer, the resultant bands structure is weaker and the formation of columnar grains along the build direction prevails [63]. A bimodal grain structure has been observed when this 90° rotation scan strategy is adopted due to the periodicity of the scanning pattern; this bimodal grain structure is made by columnar grains with a strong cubic texture, in which the orthogonal <100> axes tend to be oriented along the build direction and along the edges of the squares on the horizontal plane, and by fine grains, which are slightly elongated in the direction perpendicular to the edges of the squares and with a more random crystallographic orientation [34,65,66]. Wang et al. also [65] reported the frequent nucleation of randomly oriented grains at the solidification front that interrupts the epitaxial growth of the columnar grains of the previous layer. In accordance with the above discussion, the prevalence of the heterogeneous nucleation on the epitaxial growth leads to the formation of grains that are more confined within the thickness of the deposited layer and by the laser related features (i.e., the squared scan islands in that case).

In the current study, a bimodal grain structure was not observed, with most of the grains being columnar in shape and aligned along the build direction crossing multiple melt pools (Figure 4e,f and Figure 11). Furthermore, a marked preferential [001] orientation along the build direction was detected from the EBSD analysis (Figure 6 and Figure 8), but no preferential orientation of the other two orthogonal <100> axes was evident. The development of the grains during the SLM process was not confined to the melt pools and the laser tracks, with the laser related boundaries not being detectable in the EBSD images (Figure 5 and Figure 7). This shows the prevalence of the epitaxial growth of the columnar grains across different layers on the nucleation of new randomly oriented grains during the advancement of the solidification front.

Based on the comparison between our results and the aforementioned studies, it can be inferred that the grains which can grow across more deposition steps have a crystallographic orientation that better fits the global shape of the thermal flux field resulting from the adopted scan strategy. The repetitive scan strategies, or those which involve a periodic repetition of the scanning pattern after a small number of deposited layers, lead to a more heterogeneous and anisotropic thermal flux field, to which the grains are aligned. For example, as discussed above, the simple scan strategy without rotation selects the grains with <100> texture along the scan direction and <110> texture along the build direction, while the island scan strategy selects the grains with the <100> axes parallel to the build direction and to the edges of the squared islands. In both of these examined cases, the ideal crystallographic orientation is rigid and so few grains are expected to be selected.

The non-repeating nature of the 67° rotation scan strategy adopted for this study led to a more isotropic situation because the effects of the horizontal components of thermal fluxes (i.e., lying on the plane of the added layers) offset each other, thus only the component parallel to the build direction had a strong role in driving the growth at the length scale of the grains. The result of this scan strategy was the observed strong and quite homogeneous <100> texture along the build direction (Figure 7), but another important consequence is that the distribution of the favored crystallographic orientations was symmetric around the build direction, thus the selection of the grains was much less severe. In these conditions, the epitaxial growth of the preexisting grains prevailed over the heterogeneous nucleation of new grains and, for this reason, small randomly oriented grains at the laser related boundaries were not observed. Additionally, Thijs et al. [67] have observed how the choice of the scan strategy in the SLM process has an important effect on the competitive growth of the grains; however, they state that the rotation of the scan direction of an angle of 90° or 60° after each deposition step leads to a more severe competition, with the consequent development of a stronger texture. Furthermore, Wan et al. [63] reported a stronger texture in an Inconel 718 alloy obtained with a 90° rotation scan strategy with respect to those obtained without any rotation. We state that the 67° rotation has the effect of reducing the harshness of the competition between grains by relaxing one constraint (i.e., the angle around the build direction) on the ideal crystallographic orientation. The disagreement with the above-mentioned studies [63,67] can be explained by considering that the 90° and 60° rotation scan strategies both involve a repetition of the scan pattern after two and three layers, respectively, which are probably not enough to guarantee an effective symmetry along the build direction.

### 4.2. Development of the Intragranular Dendrites

The morphology of the solidification front is controlled by the G/V ratio [68], where G is the thermal gradient in the liquid and V is the solidification velocity. A high G/V value leads to a stable planar interface, while a low value results in the formation of cells and dendrites. The destabilization of the planar interface is related to the formation of constitutional undercooled liquid in front of it. The essential condition for this to occurs for a binary alloy under steady-state solidification is given by [58]:

(3) $$\frac{G}{V}<\frac{T_{L}-T_{S}}{D}$$

where $T_{L}-T_{S}$ is the range between the liquidus and the solidus temperatures and D is the diffusivity of the solute in the liquid. It is known from Equation (3) that, at a given value of G, a constitutional undercooling region forms in front of the solid–liquid interface when the solidification velocity exceeds a critical value. As a consequence, the planar interface becomes unstable and the observed cellular dendrites can develop and grow along the direction of the thermal gradient [58,60,69]. Wei et al. calculated the G/V value of the SLM process [61]: In each layer, the G/V value falls in the range between 20 and 100 K s mm^−2^, and a threshold value for the stabilization of the planar front is in the order of 7000 K s mm^−2^. Therefore, the columnar dendrite formation is significantly favored as observed. No evidence was found to suggest nucleation in the core of the liquid volume, far from the solid–liquid interface, or the subsequent formation of equiaxed dendrites.

The melt pool solidification is also characterized by very high cooling rates. Hooper [70] used a coaxial high-speed temperature imaging system to monitor the temperature field in the melt pool of a Ti6Al4V powder bed and reported an average thermal gradient and cooling rate of 5–20 K/µm and 1–40 K/µs, respectively. Li et al. [71] and Song et al. [72] used numerical modeling to obtain the cooling rate at different zones of the melt pool of Inconel 718 during directed energy deposition (DED) and reported an average cooling rate range of 2300–6800 K/s. The high cooling rate of the melt pool led to strong microsegregation and the development of an extremely fine subgranular cellular microstructure characterized by small dendrite arm spacing. The mean dendrite arm spacing observed in this study was in the order of 1 µm (Figure 13), which is comparable to that reported by Amato et al. [19] and other studies [16,17,20,47,53]. However, Popovich et al. [35,40] reported coarser dendrites with a dendrite arm spacing of approximately 2–3 µm; in their work they used higher laser power and lower scan speed when compared to those used this study, which favor the formation of larger dendrite sizes due to the formation of larger melt pools and thus lower cooling rates [73]. The primary dendrite arm spacing obtained in SLM process is usually much lower when compared to the DED techniques, for comparison Tian et al. observed a dendrite size of 5–7 µm with peaks of 20 µm [33].

Prominent [001] texture and fine cellular substructure along the build direction were also reported by Mostafa et al. [36], who have also adopted the 67° rotation scan strategy. In addition, morphological differences were found in the laser overlapping regions, where the authors observed a larger dendrite arm spacing and changes in the dendrite growth direction, which are explained with a different experienced cooling rate respect to the zone within the laser tracks [36]. The dendrite growth direction and size were found to be non-uniform over the entire grain also in the current study, although the observed variations seem to us more related to the laser related boundaries: Abrupt changes of 90° in the growth direction were sometimes observed at the boundaries between adjacent laser tracks (Figure 4b, Figure 9b and Figure 11b); the dendrite size changed across the arc-shaped boundaries of the melt pools (Figure 12 and Figure 14).

As discussed above, the growth direction and length scale of the dendrites are related to the direction of the heat fluxes in the melt pool and the cooling rate, respectively. Therefore, it is inferred that the aforementioned inhomogeneities are related to the dynamic temperature field that evolves during the solidification of the melt pool [60,70,71,72]. At the beginning of solidification, the local cooling rate of the liquid in contact with the solid substrate is high leading to the formation of narrow dendrites, but after this first solidification stage the cooling rate is reduced [70,72] and so a slight increase in the dendrite size occurs in the top part of the melt pool which experiences solidification last.

**Figure 23:**
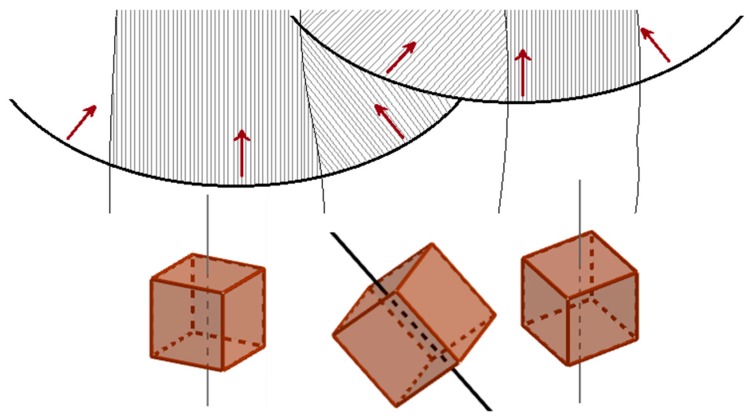
Schematic model of the solidification of columnar grains with different crystallographic orientation. The dendrites of the middle grain change their growth direction of 90° at the melt pool boundary following the local thermal gradients (red arrow) resulting in a zig-zag shape similar to that observed in Figure 9b.

A 90° rotation of the dendrite growth direction was observed to occur at the boundary of the laser tracks due to local variations in the heat fluxes direction toward the center of the melt pool. The schematic shown in Figure 23 clarifies this process. As can be seen, the dendrites of favorably oriented grains develop along their [001] axis, and can grow epitaxially in the last deposited layer through the melt pool boundary if the thermal gradient direction does not vary across it. However, the thermal gradient direction changes abruptly close to the borders of laser scans, nevertheless the dendrites can still grow epitaxially from the new layer if they are favorably oriented with respect to the new direction of the local thermal gradient (i.e., if one of their [100] or [010] axes is set at a small angle with respect to the local heat flux). Whenever this condition is satisfied, the dendrites start to grow at a 90° angle relative to the previous growth direction. This phenomenon leads to the zig-zag path of grain growth, as observed in Figure 9b. A similar zig-zag epitaxial growth of the grains across subsequent layers was observed in the deposition through DED techniques when a scan strategy of parallel scan lines with altered direction is adopted [37,61,74]. In the current study, where a powder-bed technique and a more complex scan strategy were adopted, the zig-zag growth was not a global feature, but it concerned only the grains that are coincidentally in a favorable position and with the correct crystallographic orientation, as represented in Figure 23. The 90° rotation of the dendrites at the melt pool boundaries in an Inconel 718 alloy produced through SLM is also reported by Deng et al. [75].

Theoretically, it is possible to assert that the dendrites in each grain should have the same crystallographic orientation. However, the high-resolution EBSD maps in Figure 9b shows some elongated features with slight reciprocal misorientation inside one grain that developed with abrupt change of 90° in the direction. Although the resolution of 0.71 µm/pixel in the EBSD analysis (Figure 9b) is too low to obtain a sharp orientation map at the dendrite length scale, our observations suggest that it is possible that a subgranular structure caused by slightly misoriented colonies of dendrites formed inside some grains. Intragranular dendrites with low-angle misorientation were also reported by Chlebus et al. [38] and Choi et al. [64]. Furthermore, Divya et al. [30] reported a high-resolution EBSD analysis in a single grain of SLM CM247LC Ni alloy, showing a misorientation of lower than 1° between intragranular dendrites that leads to a gradual variation of the orientation across the grain.

The intragranular misorientation can explain the features observed in the grain maps reported in Figure 5, where the largest grains were constituted by subgranular domains surrounded by low-angle boundaries. The slight misorientation between the forming intragranular dendrites led to a crystallographic orientation gradient inside the grain; then a rearrangement of the atoms occured, driven by the reduction of the misorientation energy, with consequent formation of the observed subgranular domains. The rearrangement can be triggered by the thermal cycles to which the as deposited material is subjected at each deposition of a new layer. The presence of the subgranular domains can make the unambiguous identification of the grains in the as-built material difficult.

### 4.3. Formation of the Dendrite’s Features: Microsegregation, Eutectic Phases, and Dislocations

Microsegregation and microstructural inhomogeneities in a single dendrite as shown in Figure 13b, Figure 14b and Figure 15 were due to the path of solidification during the SLM process. The high solidification rate caused the elements to distribute in the solid and remaining liquid at a ratio that reflects their own partition coefficient *k* (i.e., the ratio between the equilibrium solute concentrations in the solid and in the liquid phase; approximatively constant with the temperature). Elements with a value of *k* lower than 1 tended to segregate in the last solidifying zone (i.e., the interdendritic boundaries), while the dendrite cores remained more enriched in the elements when *k* is higher 1. In the Inconel 718 system, the alloying elements with a *k* > 1 are Ni (1.03), Cr (1.09), and Fe (1.20), while those with *k* < 1 are Nb (0.28), Ti (0.41), Mo (0.73), and Al (0.79) [76]. As expected, the EDS analysis reported in Figure 16 indicates that Nb was the most dominant segregation among all the other alloying elements due to its very low partition coefficient, large atomic radius and the consequent low diffusivity in the γ phase which prevents solute redistribution. The interdendritic liquid is progressively enriched by Nb during the formation of the primary γ phase, then the solidification ceases through two non-invariant eutectic transformations that occurs at the interdendritic boundaries [77,78,79,80]: L → L + γ + NbC and L → γ + Laves phases.

The microstructure of the dendrite observed in the current study (Figure 13, Figure 14 and Figure 15) was in good agreement with the solidification path described above. Carbides and intermetallic Laves phases are expected near and in correspondence to the interdendritic boundaries. The eutectic products were present in divorced form [38]. Carbides were identified as 25–50 nm sized blocky or rounded particles whose density increases approaching the interdendritic edges (they appear in dark contrast in the STEM images in Figure 15 and are significantly enriched by Nb). Laves phases were placed mostly at the triple points between cells on the horizontal plane (Figure 13b) and disposed along the boundaries between dendrites on the vertical plane (as shown in Figure 14b, in bright contrast in the STEM images in Figure 15 and Figure 17 as confirmed by the SAED analysis). A Laves phase is a metastable Topologically Close Packed (TCP) phase [81] with general formula (Ni,Cr,Fe)_2_(Nb,Mo,Ti). Usually, Laves phases are undesirable due to its embrittlement effect and the decrease of the availability of Nb for the formation of the γ″ strengthening phase which can results in the reduction of the mechanical properties [82]. A post homogenization heat treatment is often required to dissolve Laves phases.

Dislocations were also present due to internal stress and consequent plastic deformation caused by the high thermal gradients and consecutive thermal cycles during the SLM process [53]. Dislocations tended to accumulate at the interdendritic boundaries (Figure 15) in order to accommodate the misorientation between cells [30]. Furthermore, higher dislocation density was observed around the interdendritic second phases, in particular the relatively coarse Laves particles, suggesting that the presence of these precipitates, which are able to block the dislocation motion, contributed to the accumulation of the line defects at the interdendritic boundaries.

### 4.4. Evolution of the Microstructure during the Aging Treatment

In the collected DSC curves (Figure 18), the exothermal peaks indicate the precipitation of second phases, while the dissolution of the second phases is an endothermic phenomenon.

The first two exothermal peaks were due to the precipitation of the strengthening γ′ and γ″ phases, respectively. ENDO1 was due to the dissolution of the previously formed γ′ and γ″ precipitates. EXO3 was related to the formation of the δ phase.

The last endothermic peak ENDO2 was caused by the dissolution into solid solution of the previously formed δ phase and the partially dissolution of the pre-existing metastable phases, as mainly the Laves compounds observed in the as-built state.

The variation in the lattice parameters after thermal treatment was ascertained from the XRD analysis (Figure 20 and Figure 21). The lattice parameter increased after the solution step because of the greater amount of solute in the γ matrix, then it reduced during the aging because of the precipitation of γ′, γ″, and δ second phases. Increasing aging duration led to higher levels of solute transfer from the solid solution to the second phases.

Although the γ′ particles after aging at 565 °C are hardly visible at the FESEM images (Figure 22), the DSC analysis (Figure 18), the Vickers microhardness measurements (Figure 19), and the lattice shrinkage (Figure 21) detected through XRD indicate the formation of this phase at this aging temperature.

The greatest contribution to the hardness came from the γ″ formation. At 740 °C the discoidal γ″ particles were formed, preventing the formation of δ phase. The loss of Vickers microhardness after an over aging of 24 hours was likely due to coarsening of the strengthening phases [9,83,84] and by a reduction of the solid solution strengthening caused by the Nb dissolved in the γ matrix.

At 800 °C the δ phase started to form firstly at the grain boundaries, while γ″ discoidal precipitates formed inside the grain and undergo rapid coarsening. The stacking faults of γ″ are nucleation sites for the δ phase [85,86]; therefore, the presence of γ″ particles favor the formation of intragranular δ plates after long aging exposure. The γ″ and δ phase have the same Ni_3_Nb stoichiometry, but δ is the thermodynamically stable form; therefore, γ″ transforms progressively into δ during aging with consequent reduction in the strengthening level [87,88]. Furthermore, the δ phase is usually unfavorable because of its plate morphology that causes stress concentrations, although it is sometimes reported as beneficial for creep resistance [86,89].

Consistent with the DSC analyses, no trace of γ′ and γ″ were detected at the FESEM after aging at 870 °C, with the rapid formation and growth of the δ plates occurring both at the grain boundaries and inside the grains. The intragranular δ plates were oriented in a regular pattern relative to each other based on a parallelepiped grid due to the well-known crystallographic relationship between the δ phase and the γ matrix [51,90,91]: ${\left(010\right)}_{\delta }∥{\left\{111\right\}}_{\gamma },{\left[100\right]}_{\delta }∥{\langle 1\bar{1}0\rangle }_{\gamma }$.

The δ precipitates provided a strengthening of the alloy, which is sufficient to recover the decrease in hardness caused by the solution annealing step. However, the hardness level was equal or even slightly lower than the one measured for the as-built sample.

## 5. Conclusions

The microstructure of as-built SLM Inconel 718 superalloy was deeply investigated at different length scales following an optimization study of processing parameters. The as-built microstructure was characterized by high complexity, which can be described at different length scales. A systematic study of the microstructure of as-built SLM Inconel 718 was carried out and the following main features were detected:

- Length scale from 10^−3^ to 10^−4^ m: Laser related features, columnar grains developed mainly along the build direction and not confined within the melt pools or laser tracks, and presence of subgranular domains separated by low-angle boundaries. The bimodal grain structure, which is usually reported when a periodic scanning strategy is adopted, was not observed in the current study. The 67° rotation scan strategy has a role in impeding the formation of the bimodal grain structure because it removes one constraint in the selection of the most favorably oriented grains; therefore, it is assumed to reduce harshness of the competitive growth and thus to favor epitaxial growth at the expense of heterogeneous nucleation;
- Length scale from 10^−4^ to 10^−5^ m: Predominant <100> crystallographic texture of the grains and substantial isotropy of the crystallographic orientation around the build direction. The lack of observable texture on the horizontal plane is further evidence of the less severe selection of the growing grains;
- Length scale from 10^−5^ to 10^−6^ m: Columnar intragranular dendrites mainly oriented along the build direction or grown following a zig-zag path along the melt pools due to abrupt changes of 90° in the growth direction. Contrary to what was found at the grain length scale, the microstructure at the length scale of dendrites was affected by the laser related boundaries, with abrupt changes in growth direction and dendrite size observed due to the nonuniform solidification conditions and the complex thermal field in the melt pool;
- Length scale from 10^−6^ to 10^−8^ m: Microsegregation of the alloying elements inside the dendrite and presence of extremely fine particles (i.e., carbides and Laves phases) and dislocations at the interdendric boundaries.

Based on the characterization reported in this paper, it can be concluded that the as-built microstructure is not suitable for an immediate application of the material because of the observed heterogeneities, the microsegregation of the alloying elements, and the uneven distribution of a large amount of brittle precipitates. Therefore, a post-process heat treatment is required to correct the microstructure.

The temperature ranges at which the most important precipitation and solutioning phenomena of the second phases can occur, and their effect on the microhardness, were determined in this work. The formation of γ′ particles at 565 °C resulted in a slight increase in hardness, with the peak hardness being reached at 740 °C because of large precipitation of discoidal γ″; however, these precipitates underwent coarsening after prolonged aging and tended to transform to plate-like δ phase with consequent decrease in hardness. At the interdendritic boundaries the small eutectic carbides persisted after the aging process and the precipitation of γ″ was more concentrated along them because of the locally high Nb content.

This work provides a complete framework of the Inconel 718 microstructure in the as-built state following the SLM process and how it can be modified through thermal treatment, and it can be used as a base for the development of a post-process heat treatment cycle specifically designed for the specifications required by the final application.

## Figures and Tables

**Table 1:** Chemical composition in wt.% of the Inconel 718 EOS GmbH (Krailling, Germany) powders used in this work [44].

**Ni**	**Cr**	**Nb**	**Mo**	**Ti**	**Al**	**Fe**
50–55	17–21	4.75–5.5	2.8–3.3	0.65–1.15	0.2–0.8	balance
**Co**	**Cu**	**C**	**Si + Mn**	**P + S**	**B**	
<1	<0.3	<0.08	<0.35	<0.015	<0.006	

**Table 2:** Investigated combinations for the optimization study of the process parameters and relative volumetric energy density (VED) values.

Sample	Power [W]	Scan Speed [mm/s]	Hatching Distance [mm]	VED [J/mm^3^]
1	175	600	0.09	162.04
2	195	600	0.09	180.56
3	175	900	0.07	138.89
4	175	1200	0.11	66.29
5	175	1200	0.07	104.17
6	185	600	0.09	171.30
7	185	1200	0.11	70.08
8	185	900	0.07	146.83
9	175	1200	0.09	81.02
10	185	900	0.11	93.43
11	195	900	0.07	154.76
12	185	600	0.07	220.24
13	195	600	0.07	232.14
14	195	1200	0.09	90.28
15	175	600	0.07	208.33
16	195	1200	0.11	73.86
17	195	900	0.09	120.37
18	185	900	0.09	114.20
19	185	1200	0.07	110.12
20	185	1200	0.09	85.65
21	175	900	0.09	108.02
22	175	600	0.11	132.58
23	195	600	0.11	147.73
24	195	1200	0.07	116.07
25	185	600	0.11	140.15
26	195	900	0.11	98.48
27	175	900	0.11	88.38

**Table 3:** Optimized selective laser melting (SLM) parameters.

Parameter	Value
Laser power (W)	195
Scan speed (mm/s)	1200
Hatching distance (mm)	0.09
Spot size (μm)	100
Layer thickness (μm)	20

**Table 4:** List of the post-process heat treatment recipes and total aging exposure time applied for the study on the aging response of selective laser melting (SLM) Inconel 718.

Sample	Heat Treatment Recipe	Total Aging Exposure
Disk 1	1065 °C/2 h, 565 °C/4 h	4 h
	+565 °C/20 h	24 h
Disk 2	1065 °C/2h, 740 °C/2 h	2 h
	+740 °C/6 h	8 h
	+740 °C/16 h	24 h
Disk 3	1065 °C/2 h, 800 °C/4 h	4 h
	+800 °C/20 h	24 h
Disk 4	1065 °C/2 h, 870 °C/4 h	4 h
	+870 °C/4 h	8 h
	+870 °C/16 h	24 h
